# Two-Dimensional Periodic Nanostructure Fabricated on Titanium by Femtosecond Green Laser

**DOI:** 10.3390/nano10091820

**Published:** 2020-09-12

**Authors:** Yi-Hsien Liu, Shu-Chun Yeh, Chung-Wei Cheng

**Affiliations:** Department of Mechanical Engineering, National Chiao Tung University, No. 1001, Ta Hsueh Road, Hsinchu 300, Taiwan; kevin778899.me07g@nctu.edu.tw (Y.-H.L.); obey1227.me07g@nctu.edu.tw (S.-C.Y.)

**Keywords:** femtosecond laser, nanostructures, LIPSS, LSFL, HSFL

## Abstract

Laser-induced periodic surface structures (LIPSS) is the sub-wavelength periodic nanostructure, which is generally generated by the femtosecond laser. There are two kinds of LIPSS, low spatial frequency LIPSS (LSFL) and high spatial LIPSS (HSFL), and the period size is close and less than half of the laser wavelength, respectively. Fabrication of two-dimensional (2D) LSFL and HSFL on a titanium surface with a linear-polarized femtosecond green laser beam (wavelength 515 nm) and cross-scanning strategies is demonstrated in this study. Four types of LIPSS structures are obtained by controlling the laser fluence, irradiated pulses, and cross-scanning strategies: 1D-LSFL perpendicular to laser polarization with a period of 300–360 nm, 1D-HSFL parallel to laser polarization with a period of 55–75 nm, 2D-LSFL dot-like structures with a period ~200 nm, and 2D-HSFL net-like structures with a period of 50–100 nm.

## 1. Introduction

LIPSS (laser-induced periodic surface structures) has attracted lots of attention because it can be simply generated on metal, semiconductor, and dielectric material by a single-pass laser beam [1]. There are two different types of LIPSS that can be generated on the material surface, depending on different laser fluence inputs. One is LSFL (low spatial frequency LIPSS, period > λ/2,), which can be generated when the fluence is near the ablation threshold and the orientation is usually perpendicular to the polarization. The other one is HSFL (high spatial frequency LIPSS, period < λ/2) in which the orientation is usually parallel to the polarization and the fluence input is relatively lower than LSFL. Titanium is an important material in many fields because it has lots of advantages, such as high corrosion, temperature resistance, and good biocompatibility. Therefore, there are some LIPSS studies [2,3,4,5,6], which are aimed at changing the physical and chemical properties of Ti for different niche applications, e.g., bio- and dental implant, tribological, and low-reflective surfaces.

In the LSFL regime, Tsukamoto et al. generated the LSFL and periodic microstructures (few µm) on titanium by different laser fluence and different pulse numbers. They found that the one-dimensional 1D-LSFL is perpendicular to the polarization and the periodic microstructures are parallel to the polarization [7]. Lehr et al. generated the micro/nano hybrid structures on titanium by different scanning overlapping ratio [8]. Gemini et al. used a dual-pulse method with a time delay of 160 fs to generate the surface structures on the titanium and generate1D-LSFL on the surface [9]. Gnilitskyi et al. proposed that the 1D-LIPSS can be manufactured on different metal surfaces, with a high scanning speed of 3 m/s [10].

On the other hand, in the HSFL regime, some groups used different wavelengths of the laser to irradiate the titanium [11,12,13,14,15]. Bonse et al. generated some 1D-HSFL structures (period ~λ/10) on titanium by Ti:Sapphire laser (790 nm). They proposed that the oxide layer which is generated by repetitive irradiation can induce the 1D-HSFL [11,12]. In addition, Kirner et al. used different chemical analyses to analyze the chemical properties of LSFL and HSFL. They found that the 1D-LSFL and 1D-HSFL with an oxide layer of 200 nm and 10 nm are formed, and they consisted of TiO_2_ and partially crystallized Ti_2_O_3_ [13]. In a related study, Nathala et al. found that the period of 1D-HSFL is varied from 60 nm to 120 nm with laser fluence 32–44 mJ/cm^2^ [15]. They also proposed that the Gaussian beam profile is not suitable for generating large-area 1D-HSFL because of the intensity distribution. Li et al. controlled the fluence which is below the ablation threshold to irradiate the titanium. They found that the Ti*_x_*O*_y_* is first formed on the titanium surface. These oxides could enhance the third-harmonic generation and form the HSFL [14].

Recently, some studies used different methods (controlling the polarized direction, scanning strategy, and dual pulse) to generate the two-dimensional (2D) micro/nano structures. Hermens et al. generated a large-area 2D-LSFL on the nitride steel by changing the different directions of polarization. They found that when the relative angle of the second irradiation is larger than the first irradiation (>20°), the diamond-shaped structures can be generated on the overlapping area [16]. Kobayashi et al. used a cross-scanning strategy to generate 2D nanodot structures with the size around 800 nm on stainless steel [17]. Fraggelakis et al. used the dual-pulse method to generate the surface structures on the nickel. They found that when the delay time of the second pulse is around 15–25 ps, the 2D sub-100 nm holes can be generated on the surface [18]. However, there is no published research that is focused on generating 2D-LSFL and 2D HSFL by cross-scanning strategies on the titanium.

In this research, the top-hat femtosecond laser beam with a green wavelength was used to generate the LSFL and HSFL on the titanium surface. The cross-scanning strategies with different scanning parameters were used to generate the 2D structures. The surface morphology generated by different methods was investigated.

## 2. Materials and Methods

**Figure 1:**
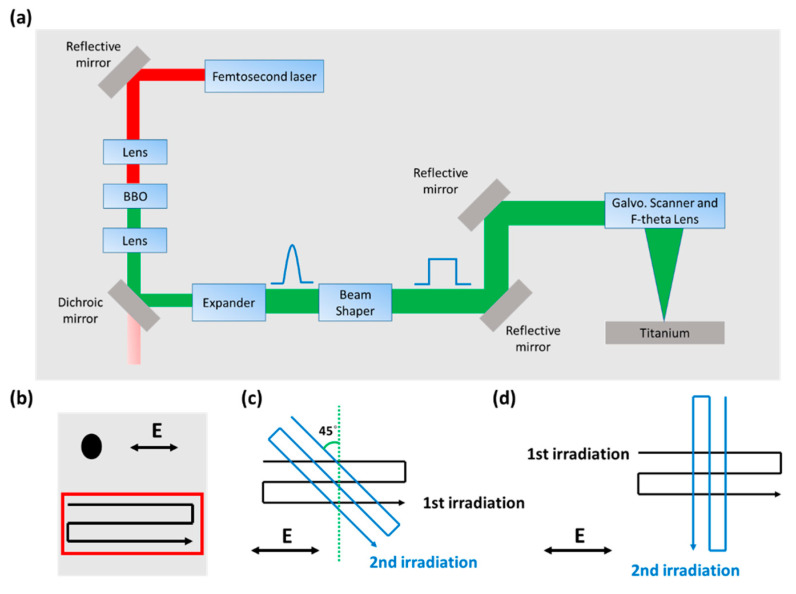
(**a**) Schematic of the experiment setup; (**b**) percussion irradiation and area patterning process; (**c**) Case I and (**d**) Case II scanning strategies to fabricate the structures.

Mechanically polished titanium (grade 1) was nanostructured in the air with a femtosecond fiber laser machining system Figure 1a that had an infrared femtosecond fiber laser (KASMORO, mRadian Inc, Hsinchu, Taiwan), with a wavelength of 1030 nm, pulse duration of 420 fs, beam diameter of 2.4 mm (1/e^2^), a repetition rate of 100 kHz, and an average laser power of 3 W. The laser beam was linearly polarized and passed through the Beta-Barium Borate (BBO) crystal to convert the laser beam with wavelength 515 nm and beam diameter 1.7 mm (1/e^2^). The laser beam was then expanded to 3.4 mm by an expander (magnification 2×) and converted to a top-hat profile by a beam shaper (FBS2-50-532, TOPAG Lasertechnik GmbH, Germany). The transmitted beam passed through a series of mirrors, subsequently entering a galvanometric scanner and focused on the titanium surface by F-theta lens with a focal length of 165 mm. The focal square top hat’s width *W_top_* (at 1/e^2^) was determined to be approximately 50 µm by *W_top_* = *2λf/D_gau_*, where *λ* is laser wavelength, *f* is the focal length of focusing lens, and *D_gau_* is Gaussian beam diameter.

The schematic drawing of the laser machining process to fabricate the different LIPSS structures is shown in Figure 1b–d. In Figure 1b, the percussion irradiation and area patterning process was conducted to generate the LSFL and HSFL with different laser fluence and the number of pulses (*N*). In Figure 1c,d, two different types of cross-scanning strategies were used to investigate the structures generated. For the first irradiation, the scanning direction was in the horizontal direction and parallel to the laser polarization. Then, the sample was rotated at 45° (named as Case I) and 90° (named as Case II) to conduct the second irradiation.

The surface characteristics of the structures were measured by scanning electron microscopy (SEM, Hitachi Su-8010, Tokyo, Japan), equipped with an EDS module. Fourier transform analyses were performed using MATLAB software (Natick, MA, USA) to analyze the LIPSS period. The UV-Vis-NIR spectrophotometer (Hitachi U-4100, Tokyo, Japan), with integrating sphere was used to measure the reflectance of fabricated surfaces (5 × 5 mm^2^ in size). The detector switching wavelength was 850 nm, the light source switching wavelength was 340 nm, and the wavelength steps of 2 nm was used.

## 3. Results

### 3.1. 1D LIPSS Structures

**Figure 2:**
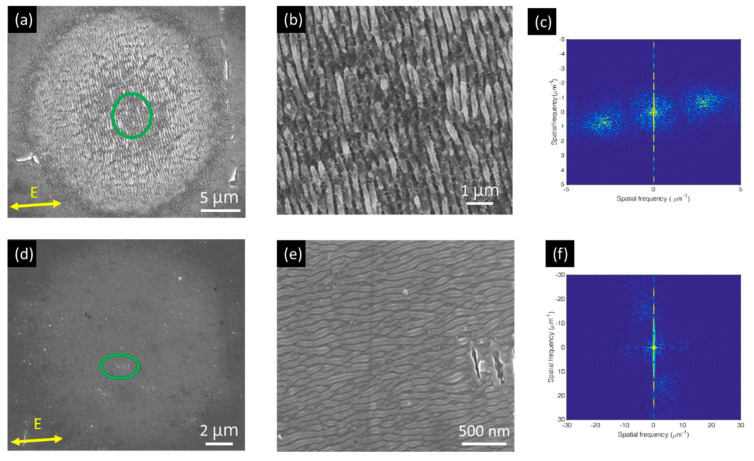
SEM images of laser-induced periodic surface structures (LIPSS) structures obtained by a stationary laser beam irradiation with different fluence and number of pulses: (**a**) 40 mJ/cm^2^, 125 pulses; (**d**) 22 mJ/cm^2^, 75 pulses; (**b**,**e**) are magnified images from the green circle of (**a**,**d**), respectively. The corresponding FFT images are shown in (**c**) and (**f**).

Figure 2 shows SEM images of two LIPSS structures observed on titanium surface after irradiation by a stationary laser beam with different laser fluence (40 mJ/cm^2^, 22 mJ/cm^2^) and the number of pulse *N* (125 and 75). As seen in Figure 2a, an irradiated area with a diameter (*D_e_*) 25 µm was obtained. The magnified image (Figure 2b) indicates that periodic-like 1D-LSFL with periods of around 330 ± 30 nm were formed, which is slightly lower than the laser wavelength (515 nm). The orientation of LSFL is perpendicular to the polarization (E) of the laser beam. When the fluence was decreased to 22 mJ/cm^2^ and *N* = 75, as shown in Figure 2d, an area with a diameter of 15 µm was obtained. Figure 2e shows that the periodic-like 1D-HSFL parallel to the polarization (E) under periods of around 65 ± 10 nm were formed, which is about seven times smaller than the laser wavelength (515 nm).

**Figure 3:**
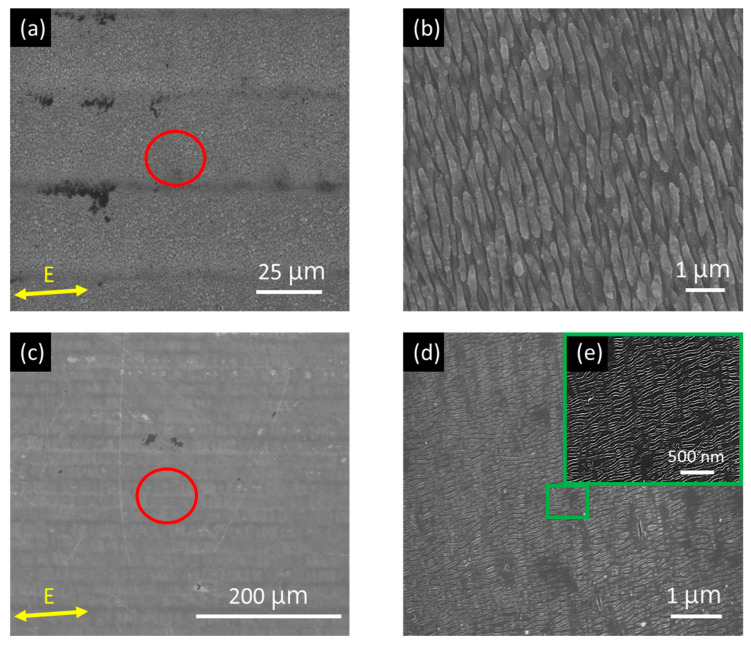
SEM images of area pattern with LIPSS structures obtained by scanning speed 20 mm/s and different fluence: (**a**) 40 mJ/cm^2^ (**c**) 22 mJ/cm^2^; (**b**,**d**) are magnified images of the red circles in (**a**,**c**), respectively; (**e**) shows magnified images of the green square in (**d**).

Figure 3 shows the SEM images of area pattern made by the zigzag scanning (see Figure 1b) with scanning speed of 20 mm/s, laser fluence (40 mJ/cm^2^ and 22 mJ/cm^2^) and hatch distance (27 µm and 18 µm), respectively. The scanning speed and hatch distance were determined by the *N* and *D_e_*, respectively. The scanning speed V_s_ is estimated by V_s_ = *D*_e_*f_rep_*/*N*, where *f_rep_* is the laser repetition rate. For example, when *D*_e_ = 25 µm (see Figure 2a), *f_rep_* = 100 kHz, the V_s_ is 20 mm/s for *N* is 125. As shown in Figure 3a,c, the large-area with 1D-LSFL and 1D-HSFL were generated. Enlarged images show that the period and orientation characteristics are similar to the stationary irradiation results.

### 3.2. 2D LIPSS Structures

**Figure 4:**
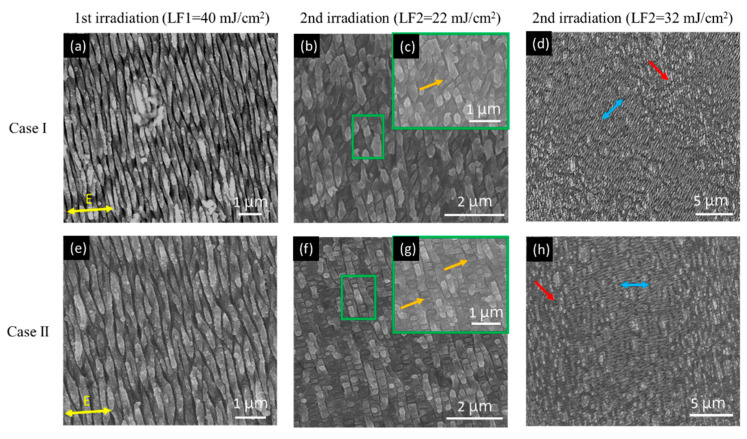
SEM images of low spatial frequency LIPSS (LSFL) surface morphology obtained by different cross-scanning strategies cases and laser fluence: (**a**–**d**) Case I. (**e**–**h**) Case II; (**c**,**g**) are magnified images of the green squares in (**b**,**f**), respectively.

Figure 4 shows the SEM images of different LSFL structures made by the Case I and Case II scanning strategies (see Figure 1c,d), respectively. Here, the laser fluence of first and second irradiation are named LF1 and LF2, respectively. The scanning speed for the first and second irradiation is 20 mm/s. The hatch distances for the first and second irradiation are 27 µm and 18 µm, respectively. As shown in Figure 4a,e, after the first irradiation by LF1 = 40 mJ/cm^2^, the fabricated 1D-LSFL is similar to Figure 3b. After the second irradiation by LF2 = 22 mJ/cm^2^, the dot-like structures with feature size 200 nm were formed, as shown in Figure 4c,g (marked by orange arrows). By changing the scanning direction, the different types of dot-like structures can be generated. However, when the LF2 increased to 32 mJ/cm^2^, the LSFL generated by the first irradiation was partially rewritten, and the new LSFL (marked by blue double-arrows) by the second irradiation was created, as shown in Figure 4d,h. However, the edge of the flat-top laser beam still had some low laser intensity, and few dot-like structures (marked by red arrows) were formed during the cross-scanning process.

**Figure 5:**
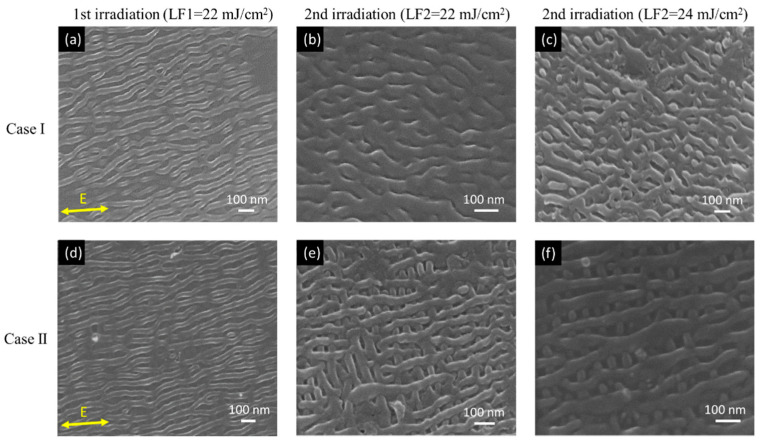
SEM images of high spatial LIPSS (HSFL) surface morphology obtained by different cross-scanning strategies, cases, and laser fluence: (**a**–**c**) Case I. (**d**–**f**) Case II. SEM tilt angle (25°).

Figure 5 shows the SEM images (tilt angle 25°) of different HSFL structures made by the Case I and Case II scanning strategies (see Figure 1c,d), respectively. The scanning speed and hatch distance for the first and second irradiation are 20 mm/s and 18 µm, respectively. As shown in Figure 5a,d, after first irradiation by LF1 = 22 mJ/cm^2^, the 1D-HSFL similar to Figure 3d are generated on the surface. After second irradiation by LF2 = 22 mJ/cm^2^, by changing the scanning direction, different 2D network-like structures with rectangular hole size <100 nm were formed, as shown in Figure 5b,e. In Figure 5e,f, it can be observed that there is a structure in the other direction, formed at the bottom of the first structure.

### 3.3. EDS Analysis

**Figure 6:**
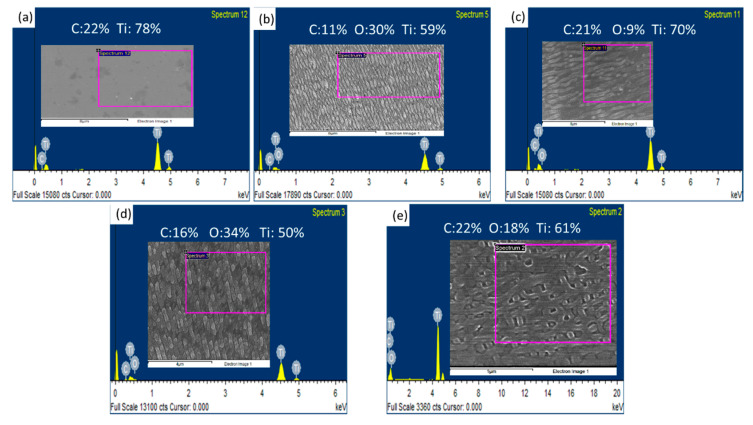
EDS analysis of the sample with/without nanostructures: (**a**) Unirradiated sample, (**b**) 1D-LSFL, (**c**) 1D-HSFL, (**d**) 2D-LSFL, and (**e**) 2D-HSFL.

EDS analysis was conducted to measure the chemical composition of the unirradiated samples and different types of LSFL and HSFL nanostructures. The acceleration voltage was set at 15 kV. The EDS spectrum and the atomic percentages of carbon (C), oxygen (O), and titanium (Ti) are shown in Figure 6 and Table 1, respectively. For the unirradiated sample, the major elements were C and Ti. The detected carbon signals can be deduced by the sample grinding. After laser irradiation, the sample is melted or ablated, and the oxygen can be detected on the nanostructured surface. The content of oxygen is highly dependent on the laser energy input and the number of irradiation. For the LSFL case, the content of oxygen in 1D-LSFL was around 30%. After the second irradiation, more oxygen with 34% could be detected on the surface. The phenomenon also can be found in the HSFL case. The content of oxygen was around 9% in 1D-HSFL and the value increased to 18% after the second irradiation. The results mentioned above indicate that the oxide layer was formed on the femtosecond laser nanostructured sample surface. The influence of these oxide layers on the formation of LIPSS will be discussed in the next section.

### 3.4. Reflectance Analysis

**Figure 7:**
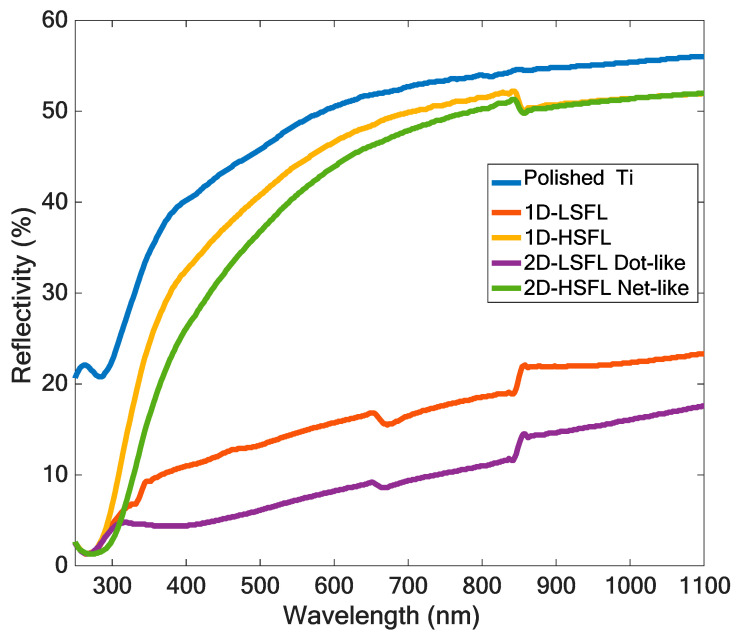
Reflectivity spectrum of the different laser-irradiated structures on Ti.

Figure 7 shows the measured reflectivity of laser-irradiated surfaces with different types of structures in wavelengths of 240–1100 nm. Compared with the polished (unirradiated) sample, the value of the sample with laser-induced structures were all decreased, and the 2D-LSFL dot-like structure had a maximum reduction. For 1D structures, the reflectivity of 1D-LSFL was below 20%, and the 1D-HSFL was slightly decreased than the polished sample. For 2D structures, the reflectivity of the 2D-LSFL dot-like structure was below 15%, and the 2D-HSFL network-like structures were similar to the 1D-HSFL. For the 1D and 2D LSFL, there are two step-like peaks could be found in 350 nm and 650–850 nm. The first step-like peaks can be deduced by the light source switching occurs. The second peaks can be deduced that the surface resonance occurred in this wavelength range. E.C. Landis et al. also published similar results [19].

## 4. Discussion

Table 2 shows the comparison of the references of LIPSS on Titanium. In the 1D-LSFL, when the laser wavelength is shorter, the structure period is smaller, and they are all perpendicular to the laser polarization direction. In the 1D-HSFL, it can be observed that the structure period has a low correlation with the wavelength, and the period is in the range of 50–150 nm, and they are all parallel to the laser polarization direction. For 2D LSFL and HSFL cases, the structure feature size is about 50–200 nm.

The formation of 1D-LSFL can be explained by the surface plasmon polarition (SPP) theory [20,21]. When metals are irradiated by a linearly polarized laser beam, the period of the 1D-LSFL is given by [22]:

(1) $$\Lambda_{LSFL}=\frac{\lambda }{\beta \pm \sin\theta_{\mathrm{inc}}},\;\beta =\sqrt{\frac{\left|\varepsilon_{1}\right|}{\left|\varepsilon_{1}\right|-1}}$$

where *λ* is the laser wavelength, *θ*_inc_ the laser incident angle, and *ε*_1_ is the real part of the relative permittivity. At room temperature, the dielectric parameter of the titanium is −4.8562 + 15.549i, Λ is calculated to be 459 nm at *λ* = 515 nm, and ~1.2 times higher than the experimental period 372 nm, since *ε*_1_ may be increased when the metal is heated by a high-intensity ultrafast pulsed laser [22].

For the research on the dynamic optical response of metallic materials under ultrafast laser irradiation, simulation and experimental comparisons have been made in related literature. For Cu, Winter et al. [23] used a simulation model (Drude-critical points model) to obtain the dynamic optical parameter and compared it with experimental transient optical properties. Chang et al. [24] used a two-temperature model (TTM) with periodic energy distribution and a Drude-critical points optical model that predicts the LIPSS dynamic optical properties. Recently, Gnilitskyi el al. [10] used a TTM and a Drude model to investigate the dynamic optical properties of Ti by femtosecond laser (1030 nm) and found that *ε_1_* increases during laser irradiation. The current study used a TTM and a modified Drude-critical point (DCP) model to investigate the Λ*_LSFL_* of Ti by femtosecond laser (515 nm). Refer to Supplementary Material for a detailed simulation model.

**Figure 8:**
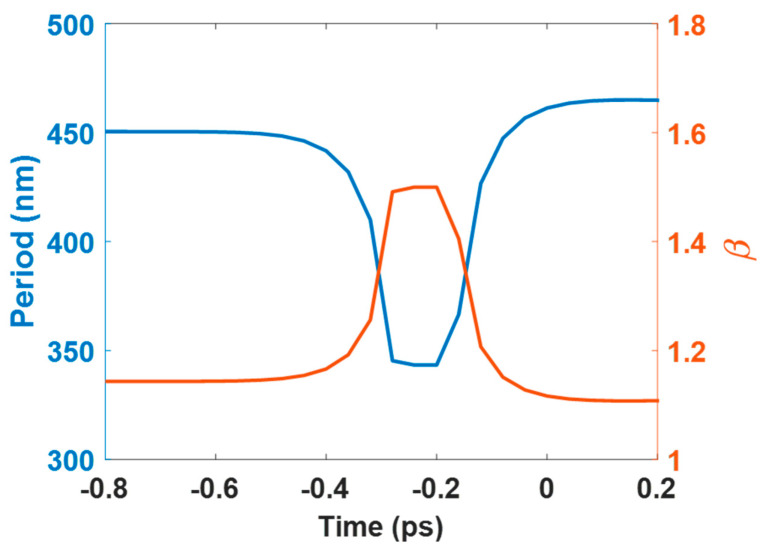
Time dependence of the calculated LSFL period and *β* for a single femtosecond laser pulse (515 nm, 400 fs) with fluences 40 mJ/cm^2^.

Figure 8 presents the time dependence of the calculated LSFL period, Λ*_LSFL_*, and *β,* for a single femtosecond laser pulse (515 nm, 400 fs), with fluences 40 mJ/cm^2^. It is obvious that the calculated period decreases during the laser irradiation, reaches the minimum ~340 nm, during the laser irradiation, and then increases as time prolongs. Note that *β* becomes zero when |ε_1_| = 0. To avoid this irrational phenomenon, the value of *β* is assumed to be 1.5 when the calculated *β* is equal to zero.

The formation of 1D-HSFL can be explained by the titanium oxide enhanced third-harmonic generation [14] and cavitation instabilities [25]. The titanium oxide generated under a repetitive laser irradiation process would enhance third-harmonic generation (THG) at the oxidized interface within the femtosecond laser beam [14,26], that is, λ/3*n*, where λ is the wavelength of the incident laser and *n* is the refractive index of the titanium oxide material. As shown in Figure 6, the 1D-HSFL has 8.56% oxygen, which means that the titanium oxide is generated during the LIPSS process. In our experiments, *n* = 1.67 of the titanium oxide (dielectric constant ε = 2.79 + 0i) [12], the nanostructures period is calculated to be 103 nm, which is close to the experimental data ~70 nm shown in Figure 2d.

However, the cavitation instabilities during femtosecond laser irradiation is another important mechanism of 1D-HSFL generation [25]. The laser would generate a shockwave and induce the recoil pressure inside the material. The recoil pressure would push the thin molten material above the surface and present protrusion structures [18,27]. For the formation of 2D nanostructures, it is speculated that in the second irradiation, due to the sub-surface cavitation [25], there will be an effect under the first structure, causing the first structure expanded above the initial position, and then forming 2D-HSFL net-like structures, as shown in Figure 5e,f. Besides, it can be deduced that the reason for generating 2D-LSFL dot-like structures lies in the fact that the HSFL generated by second irradiation cover the origin LSFL and cut the LSFL to dot-like structures.

## 5. Conclusions

This study reported a process of fabricating 2D-LSFL and 2D-HSFL nanostructures on the titanium surface by top-hat femtosecond green laser beam. The experimental results have demonstrated that, with laser fluence, 40 mJ/cm^2^, and a number of 125 pulses irradiation, 1D-LSFL structures perpendicular to the laser polarization, under an average period of 330 ± 30 nm, were formed, which is slightly lower than the laser wavelength (515 nm). When the laser fluence was decreased to 22 mJ/cm^2^, 1D-HSFL structures parallel to the laser polarization, under an average period of 55–75 nm, were formed; they are about seven times smaller than the laser wavelength. After second irradiation on the first fabricated 1D-LSFL and 1D-HSFL structures by cross-scanning strategy, dot-like 2D-LSFL (feature size ~200 nm) and network-like 2D-HSFL structures (square hole size < 100 nm) were formed. The dot-like 2D-LSFL structures have an obvious decrease in reflectance. In conclusion, the proposed technique provides a flexible means of fabricating different nanostructures required in a wide range of niche applications.

## Figures and Tables

**Table 1:** Atomic percentages of sample with/without nanostructures.

Sample	Carbon	Oxygen	Ti
Unirradiated	22	0	78
1D-LSFL	11	30	59
1D-HSFL	21	9	70
2D-LSFL	16	34	50
2D-HSFL	21	18	61

**Table 2:** Comparison of the references of LIPSS on Titanium.

Wavelength λ (nm)	Type	LIPSS Period (nm)	LIPSS Orientation to E	Fluence (mJ/cm^2^)	Number of Pulses *N*	Reference
1030	1D-LSFL	737	Perpendicular	590	2	[10]
800	1D-LSFL	460–665	Perpendicular	40–120	36	[15]
790	1D-LSFL	405–800	Perpendicular	90–350	1–1000	[12]
515	1D-LSFL	300–360	Perpendicular	40	125	This work
400	1D-LSFL	300	Perpendicular	48	220	[15]
800	1D-HSFL	50–150	Parallel	30–45	220	[15]
790	1D-HSFL	65–95	Parallel	50–90	50	[12]
515	1D-HSFL	55–75	Parallel	22	75	This work
400	1D-HSFL	50–150	Parallel	30–50	220	[15]
515	2D-LSFL	200	-	40 + 22	125 + 125	This work
515	2D-HSFL	50–100	-	22 + 22	75 + 75	This work

## Supplementary Materials

The following are available online at https://www.mdpi.com/2079-4991/10/9/1820/s1, Figure S1: Calculated dielectric constant and surface reflectivity at 40 mJ/cm^2^, Figure S2: Evolution of electron and lattice temperature at 40 mJ/cm^2^. Table S1: The parameters used in two-temperature model (TTM). Table S2: The parameters used in Drude-critical point (DCP) model.
