# Supplementary material for: Two-Dimensional Periodic Nanostructure Fabricated on Titanium by Femtosecond Green Laser
Source: Nanomaterials (Basel). 2020 Sep 12;10(9):1820. doi: 10.3390/nano10091820 (PMC7559322; doi:10.3390/nano10091820)
Supplement: Supplementary file 1 [file nanomaterials-10-01820-s001.pdf]

Supplementary Materials: Two-dimensional periodic nanostructure fabricated on titanium by femtosecond green laser

Yi-Hsien Liu, Shu-Chun Yeh and Chung-Wei Cheng

S1. Modeling of the change in the optical properties

This section discusses, in detail, the model used in this research. In our previous research, a two-temperature model (TTM) with a dynamic optical model for ultrafast laser ablation of Cu was developed [1,2]. In the current research, based on the previous model, the material properties and part of the optical model were modified and used to conduct the simulation.

The two-temperature model (TTM) was used to calculate the electron and lattice temperature since the time of femtosecond laser interaction with the material is less than the coupling time between electron and lattice. Consider a bulk titanium normally irradiated by a femtosecond laser pulse on the front surface ($z = 0$). The governing equations for 1D-TTM are given by:

$$C_e \frac{\partial T_e}{\partial t} = \frac{\partial}{\partial z}(k_e \frac{\partial T_e}{\partial z}) - G(T_e - T_l) + S(z,t) \tag{S1}$$

$$C_l \frac{\partial T_l}{\partial t} = \frac{\partial}{\partial z}(k_l \frac{\partial T_l}{\partial z}) + G(T_e - T_l) \tag{S2}$$

where t is the time, T is the temperature, C, k, and G correspond to the heat capacity, heat conductivity, and electron-phonon coupling factor, respectively. The S is laser heat source term. The laser beam is propagated along with the z-axis. The subscript e and l represent the electron and lattice, respectively.

In the modeling, the K_e is considered as linear functions varied with electron temperature, $k_e = k_{e,0}T_e/T_l$ [3]. In this research, the lattice heat conductivity is neglected because of the timescale [3]. Polynomial functions adapted from [4] are used to describe C_e and G for titanium at electron temperature below 50000 K. The parameters used in TTM are listed in Table S1. For dynamic optical properties, the heat density S can be expressed as:

$$S(z,t) = 0.94 \frac{[1 - R(0,t)]F_0}{t_p} \frac{1}{\delta(z,t)} \exp[-\int_0^z \frac{1}{\delta(z,t)} d_z - 2.77(\frac{t}{t_p})^2] \tag{S3}$$

where F_0 is the laser fluence, $R(0,t)$ is the temperature-dependent reflectivity on the front surface, and t_p is the pulse duration, wihle $\delta(z,t)$ is the temperature-dependent optical penetration depth which equals the reciprocal of optical absorption rate ($1/\alpha$). The laser pulse starts from $-2t_p$, reaches its maximum value at $t = 0$, and ends at $2t_p$.

Table S1. The parameters used in two-temperature model (TTM).

Parameter (unit)	Value (Ref.)
$K_{e,0}$ (W/m K)	22 [3]
C_e (Jm^{-3} K^{-1})	Fitting from [4]
C_l (Jm^{-3} K^{-1})	2.35×10^6 [3]
G (Wm^{-3} K^{-1})	Fitting from [4]

The Drude-critical point (DCP) model was used to describe the dynamic optical properties varied during the femtosecond laser interaction with titanium. The DCP model included a classical Drude model and two Lorentz terms, as shown:

$$\varepsilon(\omega) = \varepsilon_{\infty} - \frac{\omega_p^2}{\omega(\omega + v_{eff})} + \sum_{p=1}^{2} B_p \Omega_p \left(\frac{e^{i\phi p}}{\Omega_p - \omega - i\Gamma_p} + \frac{e^{-i\phi p}}{\Omega_p + \omega + i\Gamma_p}\right) = \varepsilon_1 + i\varepsilon_2 \quad \text{(S4)}$$

where ε_∞ is the dielectric constant, ω_p is the plasma frequency, ω is the laser frequency, V_{eff} is the electron-phonon collision frequency, p is the number of oscillators, B is a weighting factor, and Ω, $\varnothing$, and Γ are energy of gap, phase, and broadening, respectively. The value we used in the DCP model are listed in Table S2. The electron-phonon collision frequency is calculated by:

$$V_{eff} = A_e T_e^2 + B_l T_l \quad \text{(S5)}$$

where $A_e T_e^2$ and $B l T_l$ denote the electron-electron and electron-ions collisions. A_e and B_l are the constant. A_e can be calculated by the published formulae: $V_{e,e} \approx \frac{E_F}{\hbar}\left(\frac{K_B T_e}{E_F}\right)$ and $V_{e,e} = A_e T_e^2$ [5] and $A_e = 1.384 \times 10^6$ after calculating A_e, we adopted the V_{eff} fitted at room temperature in visible wavelength range by [6] and deduced the $B_l = 1.097 \times 10^{13}$.

Table S2. The parameters used in Drude-critical point (DCP) model.

Parameter (unit)	Value (Ref.)
ε_∞	1.35312 [6]
ω_p (rad/s)	2.00924×10^{16} [6]
B_1	9.01823 [6]
Ω_1 (rad/s)	2.52097×10^{15} [6]
$\varnothing_1$ (rad)	−2.06436 [6]
Γ_1 (rad/s)	*$\Gamma_1 = 0.6536 \times V_{eff}$*
B_2	3.90173 [6]
Ω_2 (rad/s)	1.88081×10^{15}
$\varnothing_2$ (rad)	2.76388 [6]
Γ_2 (rad/s)	9.05493×10^{13} [6]
A_e	1.384×10^6 (Deduced)
B_l	1.097×10^{13} (Deduced)

Based on our previous research [2], for the transition metal, the electron transition effect must be considered in the DCP model to increase the accuracy. The first broadening term, Γ_1, was replaced with a temperature-dependent parameter. The Γ_1 can be described as: $\Gamma_1 = 0.6536 \times V_{eff}$. With the calculated temperature-dependent, ε_1 and ε_2, the normal refractive index n and the extinction coefficient k can be determined by Equation S6 and Equation S7.

$$n(z,t) = \left(\sqrt{\frac{\varepsilon_1 + \sqrt{\varepsilon_1^2 + \varepsilon_2^2}}{2}}\right) \quad \text{(S6)}$$

$$k(z,t) = \left(\sqrt{\frac{-\varepsilon_1 + \sqrt{\varepsilon_1^2 + \varepsilon_2^2}}{2}}\right) \quad \text{(S7)}$$

Assuming the laser irradiates normally to the sample, the temperature-dependent reflectivity (R) and absorption coefficient (α) can be determined by Fresnel equations:

$$R(z,t) = \frac{(n(z,t)-1)^2 + k^2(z,t)}{(n(z,t)+1)^2 + k^2(z,t)} \quad \text{(S8)}$$

$$\alpha(z,t) = \frac{2\omega k(z,t)}{c} \tag{S9}$$

A finite difference method was used to solve the Equation S1 and Equation S2 and obtain the T_l and T_l. In the numerical simulation, the titanium with initial temperature of 300 K was irradiated by a laser pulse of pulse duration 400 fs and a wavelength of 515 nm. The fluence is set at 40 mJ/cm^2. After simulation, the dynamic ε_1 was used to calculate the β and the Λ which are shown in the main manuscript. Figure S1 shows the calculated ε_1 and R varied with electron temperature. The time-dependent electron and lattice temperature are shown in Figure S2.

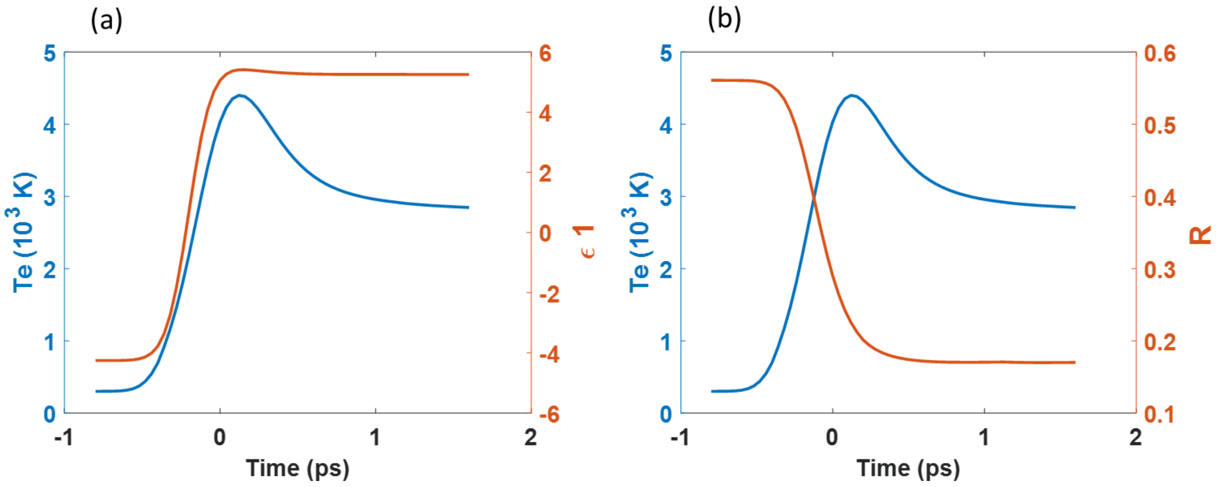

Figure S1. Calculated optical properties at 40 mJ/cm^2: (**a**) dielectric constant; (**b**) surface reflectivity.

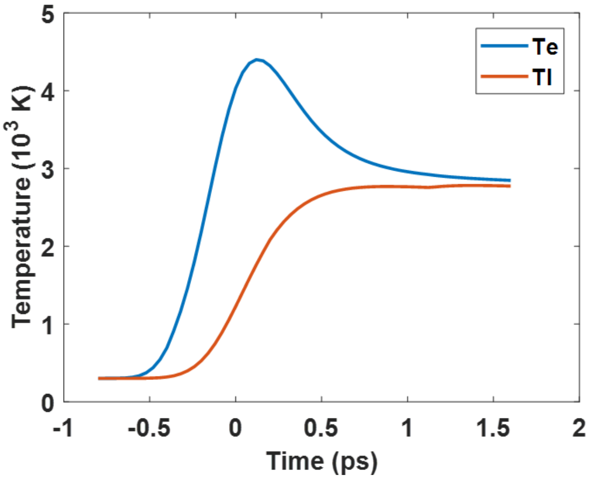

Figure S2. Evolution of electron and lattice temperature at 40 mJ/cm^2.

References

1 Chang, C.L.; Cheng, C.W.; Chen, J.K. Femtosecond laser-induced periodic surface structures of copper: Experimental and modeling comparison. *Appl. Surf. Sci.* **2019**, *469*, 904

2 Liu, Y.H.; Cheng, C.W.; Chen, J.K. Femtosecond Laser Drilling of Copper: Modeling and Experiment Comparison. *J Laser Micro nanoen.* **2020**, *15*, 25–30.

3 Wellershoff, S.S.; Hohlfeld, J.; Güdde, J.; Matthias, E. The role of electron–phonon coupling in femtosecond laser damage of metals. *Appl. Physi A* 1999, 69, S99–S107.

4 Lin, Z.; Zhigilei, L.V.; Celli, V. Electron-phonon coupling and electron heat capacity of metals under conditions of strong electron-phonon nonequilibrium. *Phys. Rev. B* **2008**, *77*, 075133

5 Fisher, D.; Fraenkel, M.; Henis, Z.; Moshe, E.; Eliezer, S. Interband and intraband (Drude) contributions to femtosecond laser absorption in aluminum. *Phys Rev. E* **2001**, *65*, 016409

6 Barchiesi, D.; Grosges, T. Fitting the optical constants of gold, silver, chromium, titanium, and aluminum in the visible bandwidth. *J. Nanophot.* **2014**, *8*, 083097
